# Estimation of *Dendrocalamus giganteus* leaf area index by combining multi-source remote sensing data and machine learning optimization model

**DOI:** 10.3389/fpls.2024.1505414

**Published:** 2025-01-15

**Authors:** Zhen Qin, Huanfen Yang, Qingtai Shu, Jinge Yu, Zhengdao Yang, Xu Ma, Dandan Duan

**Affiliations:** ^1^ College of Forestry, Southwest Forestry University, Kunming, Yunnan, China; ^2^ School of Ecology and Applied Meteorology, Nanjing University of Information Science & Technology, Nanjing, China; ^3^ College of Geography and Remote Sensing Sciences, Xinjiang University, Urumqi, China; ^4^ Information Technology Research Center, Beijing Academy of Agriculture and Forestry Sciences, Beijing, China

**Keywords:** ICESat-2/ATLAS, sentinel data, remote sensing data, sequential gaussian conditional simulation, optimization algorithm, LAI, machine learning models

## Abstract

The Leaf Area Index (LAI) is an essential parameter that affects the exchange of energy and materials between the vegetative canopy and the surrounding environment. Estimating LAI using machine learning models with remote sensing data has become a prevalent method for large-scale LAI estimation. However, existing machine learning models have exhibited various flaws, hindering the accurate estimation of LAI. Thus, a new method for large-scale estimation of *Dendrocalamus giganteus* LAI was proposed, which integrates ICESat-2/ATLAS, and Sentinel-1/-2 data, and refines machine learning models through the application of Bayesian Optimization (BO), Particle Swarm Optimization (PSO), Genetic Algorithms (GA), and Simulated Annealing (SA). First, spatial interpolation was performed using the Sequential Gaussian Conditional Simulation (SGCS) method. Then, multi-source remote sensing data were leveraged to optimize feature variables through the Pearson correlation coefficient approach. Subsequently, optimization algorithms were applied to Random Forest Regression (RFR), Gradient Boosting Regression Tree (GBRT), and Support Vector Machine Regression (SVR) models, leading to efficient large-scale LAI estimation. The results showed that the BO-GBRT model achieved high accuracy in LAI estimation, with a coefficient of determination (*R* ^2^) of 0.922, a root mean square error (*RMSE*) of 0.263, a mean absolute error (*MAE*) of 0.187, and an overall estimation accuracy (*P*_1_) of 92.38%. Compared to existing machine learning methods, the proposed approach demonstrated superior performance. This method holds significant potential for large-scale forest LAI inversion and can facilitate further research on other forest structure parameters.

## 1 Introduction

The Leaf Area Index (LAI) quantifies the total green leaf surface per unit of ground area, serving as a metric key for quantifying leaf area density within an ecosystem (Fang et al., 2019). LAI is a dimensionless parameter that exhibits considerable variability based on the growing environment, species-specific traits, leaf morphology, and other associated characteristics. The parameter is associated with numerous elements including the type of vegetation, the stage of growth, the angle of leaf orientation, the characteristics of leaf clusters, and the biomass of non-leaf components, and it is affected by the methods used for measurement. It is important for the assessment of vegetation cover and ecosystem health (Chen and Black, 1992). Consequently, the search for scientifically valid and efficient approaches to acquire large-scale spatial distribution data of vegetation LAI has become a prominent research priority in the field of forestry. While traditional direct ground survey methods can achieve high measurement accuracy, they frequently require a significant investment of both time and funds. Additionally, these methods are limited to point-scale measurements, making it challenging to obtain detailed spatial distribution data for LAI. This limitation poses significant challenges for practical applications, as it prevents researchers from effectively capturing the broader landscape variability of LAI, thereby hindering ecological assessments and management strategies.

Accurately assessing and monitoring LAI is critical, as it not only enhances the management of forest resources but also provides essential scientific insights for addressing climate change. Moreover, the continuous advancement of research techniques and data sources enables researchers to better capture and analyze variations in LAI across different temporal and spatial scales, thereby facilitating deeper exploration of related scientific inquiries and practical applications. The swift progress in satellite remote sensing technology has greatly expanded the application of LiDAR (Light Detection and Ranging), microwave, and optical remote sensing for estimating forest structural parameters, providing higher accuracy and resolution. Optical remote sensing is capable of acquiring data on the horizontal structure of forest canopies. In contrast, microwave remote sensing and LiDAR are proficient in providing information about the vertical arrangement of forest structures. Although optical remote sensing successfully addresses the limitations of conventional methods, it presents some challenges. For example, both optical and microwave remote sensing are vulnerable to topographical influences, and spectral signals may experience saturation when analyzed on a regional scale (Wulder et al., 2010). Airborne LiDAR stands as a sophisticated remote sensing technique, adept at precisely capturing vegetation structure data across various elevations, and facilitating accurate LAI inversion. Despite its advanced capabilities, the technology’s widespread application is restricted by the expensive nature of the equipment, the considerable costs involved in data collection, and its tendency to gather strip-shaped data over relatively small regions (Yan et al., 2019). Synthetic Aperture Radar (SAR) offers a significant advantage in that it can operate independently of weather conditions, allowing for continuous, all-weather monitoring of vegetation LAI. This capability enables round-the-clock data collection, making SAR particularly valuable in regions with frequent cloud cover or adverse weather. However, its broader application has been constrained by several factors, including the inherent characteristics of the sensor, the structural complexity of the vegetation canopy, and the physical properties of the surface. These limitations can introduce uncertainties in LAI estimation and reduce the overall accuracy and reliability of the data (Torres et al., 2017). As a newly developing technology in the field of active remote sensing, satellite-mounted LiDAR combines multiple existing techniques to achieve enhanced resolution and precision. This technology is capable of capturing detailed structural characteristics and surface changes of terrestrial objects with greater accuracy compared to traditional methods. Its ability to provide high-density, three-dimensional spatial data makes it particularly valuable for monitoring dynamic processes and detecting subtle variations in the Earth’s surface, offering significant advantages for environmental, ecological, and geophysical applications. This technology effectively solves the problem of “saturation” in traditional optical images and can accurately obtain detailed parameter information of the research object (Lefsky et al., 2002). ICESat-2 (Ice, Cloud, and Land Elevation Satellite-2)/ATLAS (Advanced Topographic Laser Altimeter System), provides exceptional vertical resolution and significantly improved accuracy in elevation measurements. This satellite-based LiDAR system, known for its extensive measurement range and improved resolution, has become widely utilized for precise elevation assessments and data monitoring. It is particularly effective in applications involving polar ice caps, sea ice, and forest vegetation (Narine et al., 2020).

*Dendrocalamus giganteus* is widely distributed in the southeast to southwest regions of Yunnan, known for its tall poles and wide range of applications. Thanks to its outstanding ecological adaptability and quick growth rate, it is widely appreciated in interior decor and aesthetic applications. It is often used in the creation of furnishings, ornamental items, and various construction supplies, establishing itself as a vital cash crop in Yunnan. *Dendrocalamus giganteus* is commonly used as a building material and gabion due to its sturdy material and large size, and is receiving increasing interest because of its carbon sequestration potential and environmentally sustainable characteristics, in response to the rising demand for green and eco-friendly materials. The growth of the *Dendrocalamus giganteus* bamboo sector is anticipated to enhance the earnings of local bamboo cultivators and drive regional economic development while also significantly contributing to the promotion of environmental sustainability. This growth will contribute to ecosystem conservation, enhance carbon sequestration to mitigate climate change, and protect biodiversity. Although various models have been developed over the past decade to predict the impact of climate change on biodiversity, the models have some shortcomings, such as large spatial scales and failure to consider plant adaptability (Willis and Bhagwat, 2009). As global climate change intensifies, bamboo forests, as important ecosystems, have become a research focus in terms of their response and adaptability to global climate change. Bamboo forests not only have high biodiversity, but also play an important role in carbon storage and ecosystem services (Hammerschlag et al., 2022). Therefore, the development of the *Dendrocalamus giganteus* sector represents both an engine for economic advancement and a significant measure for environmental protection.

At the present time, the research on LAI is mainly concentrated in the forest field, while the research on LAI in bamboo forests, especially *Dendrocalamus giganteus*, is relatively scarce. Therefore, the estimation of LAI for *Dendrocalamus giganteus* is conducted using ICESat-2/ATLAS alongside various remote sensing technologies, showing broad application prospects. While numerous studies have employed either traditional methods or remote sensing approaches to assess the LAI of vegetation, the majority depend solely on a single data source. This dependence may restrict the accuracy and comprehensiveness of the assessments, suggesting the need for more integrative approaches that combine multiple data sources to improve the precision of LAI estimations. This study employed the Sequential Gaussian Conditional Simulation (SGCS) technique and incorporated multiple sources of remote sensing data. Moreover, the Random Forest Regression (RFR), Gradient Boosting Regression Trees (GBRT), and Support Vector Machine Regression (SVR) models were enhanced by utilizing optimization techniques such as Bayesian Optimization (BO), Particle Swarm Optimization (PSO), Genetic Algorithms (GA), and Simulated Annealing (SA). Construct the best *Dendrocalamus giganteus* LAI remote sensing estimation model in the study area. This research offers a significant example for promoting sustainable bamboo forestry development in China, and for evaluating carbon sequestration in both forest ecosystems and bamboo plantations. The findings of this study carry substantial significance for fostering technological progress in bamboo forestry, boosting the economic value of bamboo ecosystems, and enhancing their ecological advantages.

## 2 Materials

### 2.1 Study area

**Figure 1:**
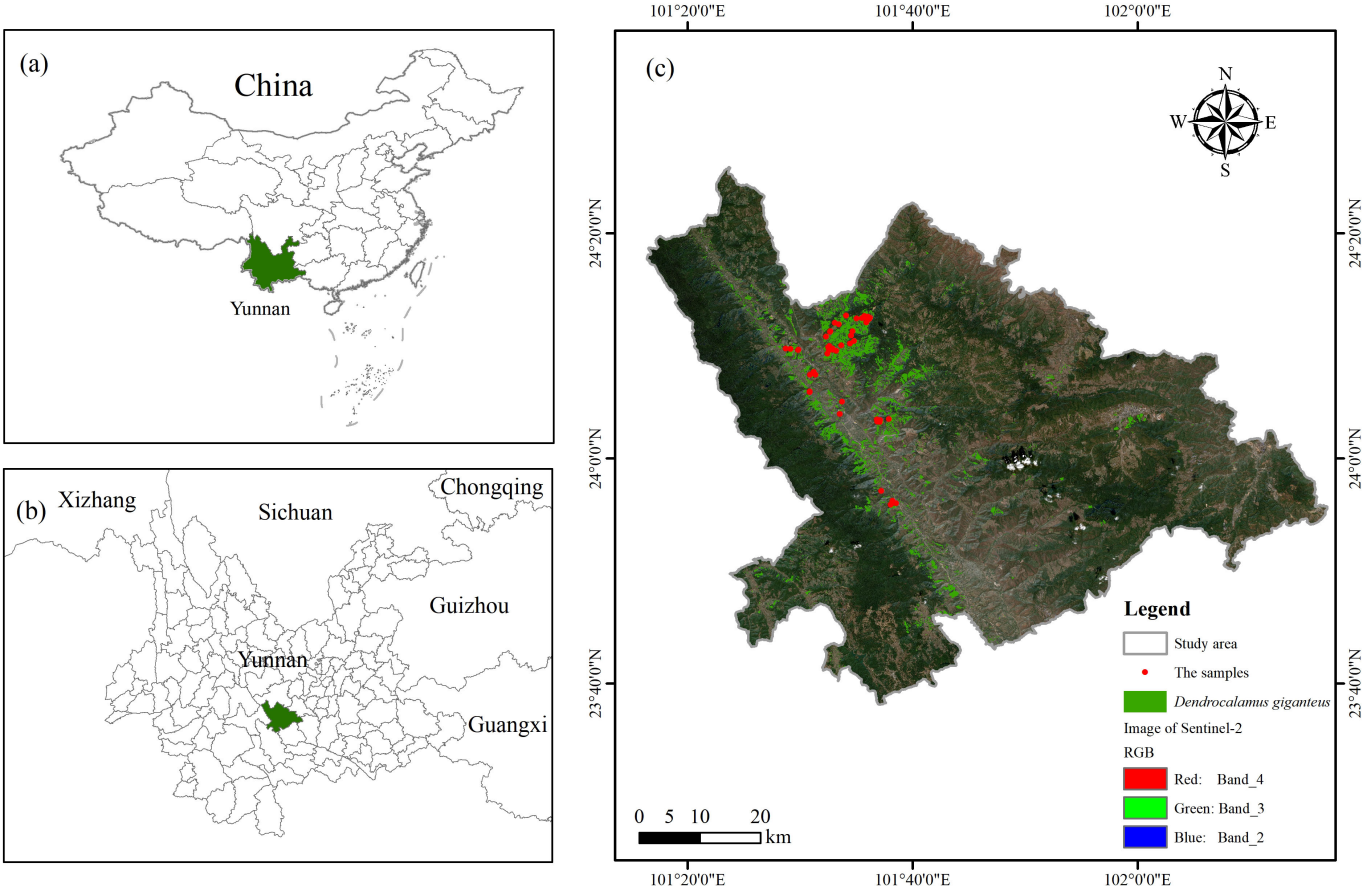
Location map of the study area. **(A)** Location of Yunnan in China; **(B)** Location of Xinping County in Yunnan; **(C)** Distribution of 51 samples in the study area.

In this study, Xinping County, located in Yuxi City, Yunnan Province, China, was selected as the study area, which is between 23°38′~24°26′N and 101°16′~102°16′E, and is geographically located as shown in **Figure 1**. Xinping County, situated southwest of Yunnan Province’s center, lies within the southwestern region of Yuxi City. Covering a total area of 4,223 km^2^, it represents 27.6% of the overall land area of Yuxi City.

### 2.2 Datasets and preprocessing

#### 2.2.1 Measured data of sample plot

This study involved the establishment of 51 circular plots, each measuring 8.5 m in radius, situated in Xinping County, within Yuxi City, Yunnan Province. This data was collected on January 8, 2024, and **Figure 1** depicts the arrangement of the sample plots. In the process of sample plot design, firstly, based on the findings from the Yunnan Province Forest Resources Planning and Design Survey, and using the Thousand Search Moment SR3 (Pro version) differential localizer (SR3, Pro version, Qianxun SI, Chengdu, Sichuan, China, https://www.qxwz.com/) in the fixed solution state of the center coordinates of the sample circle, to ensure the accuracy of the coordinates of the sample plots. In the process of data acquisition, the hemisphere photography method is adopted (Zeng et al., 2015), that is, 9 shooting points are evenly selected in the sample circle to ensure that each shooting point can cover the distribution area of the bamboo canopy, and then the fisheye lens is used to accurately shoot each point to ensure that each sample point can capture 9 fisheye photos. To guarantee accurate measurement results, it is essential that the sample site maintains suitable lighting conditions, and direct sunlight should be avoided when shooting, so as not to accurately reflect the real situation of the sample. In the process of pre-processing the fisheye photos of 51 sample plots conforming to the standard, all images were initially converted to JPG format. Subsequently, their aspect ratios were adjusted to a consistent size of 3000×4000 pixels. The images were subsequently processed using CANEYE (CANEYE, version 6.495, INRA, Paris, France, https://www6.paca.inrae.fr/can-eye/), a hemispherical image analysis software developed in MATLAB (MATLAB, version R2018a, MathWorks, Natick, MA, USA, https://www.mathworks.com/), with appropriate parameters configured. In the CANEYE software, first input the image resolution of 3000×4000, select “Create” in the “Optical Center and Projection Function”, set the “Line” value to 1228, the “Column” value to 1840, and the “P1” value to 0.0305472, observe the “Distance to the optical center (pixels)”, where Zenith (°) = P1.D (pix), then set the “COI (°)” value to 45, and in the “Fapar Computation” box, enter the values of “Day of Year” and “Latitude (%)” according to the photo acquisition time and location information at the time of acquisition, so as to perform calculation analysis. After analysis, LAI values for *Dendrocalamus giganteus* were extracted from 51 sample plots.

#### 2.2.2 Spaceborne LiDAR data

ICESat-2, the latest laser altimeter developed globally, was launched on September 15, 2018. It operates at an approximate orbital altitude of 500 km, with a 92° inclination, covering latitudes from 88°S to 88°N. The satellite completes one cycle every 91 days, tracing 1,387 orbits during each period (Markus et al., 2017). This study utilized the complete range of ATL03 and ATL08 data products, covering the entire area of investigation. These datasets were systematically acquired over a period spanning from January 2022 to August 2023, ensuring a comprehensive temporal and spatial coverage for the analysis. This dataset consists of 44 data entries, 132 tracks, and a total of 264 photonic track beams across the two categories. The data from ICESat-2/ATLAS used in this research is openly accessible and can be retrieved via the Earthdata portal at https://search.earthdata.nasa.gov/.

#### 2.2.3 Synthetic aperture radar data and optical image data

The Sentinel-1 satellite series, which includes Sentinel-1A and Sentinel-1B, are high-resolution synthetic aperture radar (SAR) satellites. Sentinel-1A was launched on April 3, 2014, followed by Sentinel-1B on April 25, 2016. Situated in a nearly polar, sun-synchronous orbit roughly 700 kilometers above Earth, these satellites deliver consistent global coverage, facilitating applications in land monitoring, disaster management, and climate change research. With a revisit interval of 12 days, the satellite ensures frequent and regular data acquisition, making it well-suited for monitoring dynamic environmental processes. Their ability to acquire data at regular intervals enhances the accuracy of long-term studies and supports informed decision-making in sustainable land use and environmental management. These two satellites operate 180 degrees apart in the same orbital plane, ensuring comprehensive coverage and data acquisition. Satellite sensors make use of C-band technology to ensure extensive global coverage and facilitate robust data transmission. These sensors operate across four distinct polarization configurations—VV, VH, HH, and HV—while maintaining a spatial resolution of 10 meters, thereby enhancing the precision and reliability of the data collected (Torres et al., 2012).

Sentinel-2 is an advanced multi-spectral imaging satellite system composed of two individual satellites, Sentinel-2A and Sentinel-2B satellites each have a 10-day revisit cycle, and provide consistent temporal coverage; however, when utilized together, they can achieve a combined revisit period of just 5 days. The Sentinel-2A satellite was successfully launched on June 23, 2015. Following this, the Sentinel-2B satellite was launched on March 7, 2017. This satellite constellation features a multispectral imager (MSI) positioned at an altitude of 786 km, which captures data across 13 spectral bands. The MSI’s swath width extends to 290 km, covering a spectral range that includes visible light, near-infrared, and shortwave infrared. The spatial resolution for different bands is as follows: B2 (blue), B3 (green), B4 (red), and B8 (Near Infrared) have a spatial resolution of 10 m; B5, B6, B7, and B8A (vegetation red edge bands), along with B11 and B12, have a spatial resolution of 20 m; and B1, B9, and B10 have a spatial resolution of 60 m (Drusch et al., 2012). The remote sensing data obtained from Sentinel-1 and Sentinel-2 utilized in this research can be freely accessed through the Google Earth Engine (GEE, https://earthengine.google.com/) platform.

## 3 Methods

### 3.1 Research design

**Figure 2:**
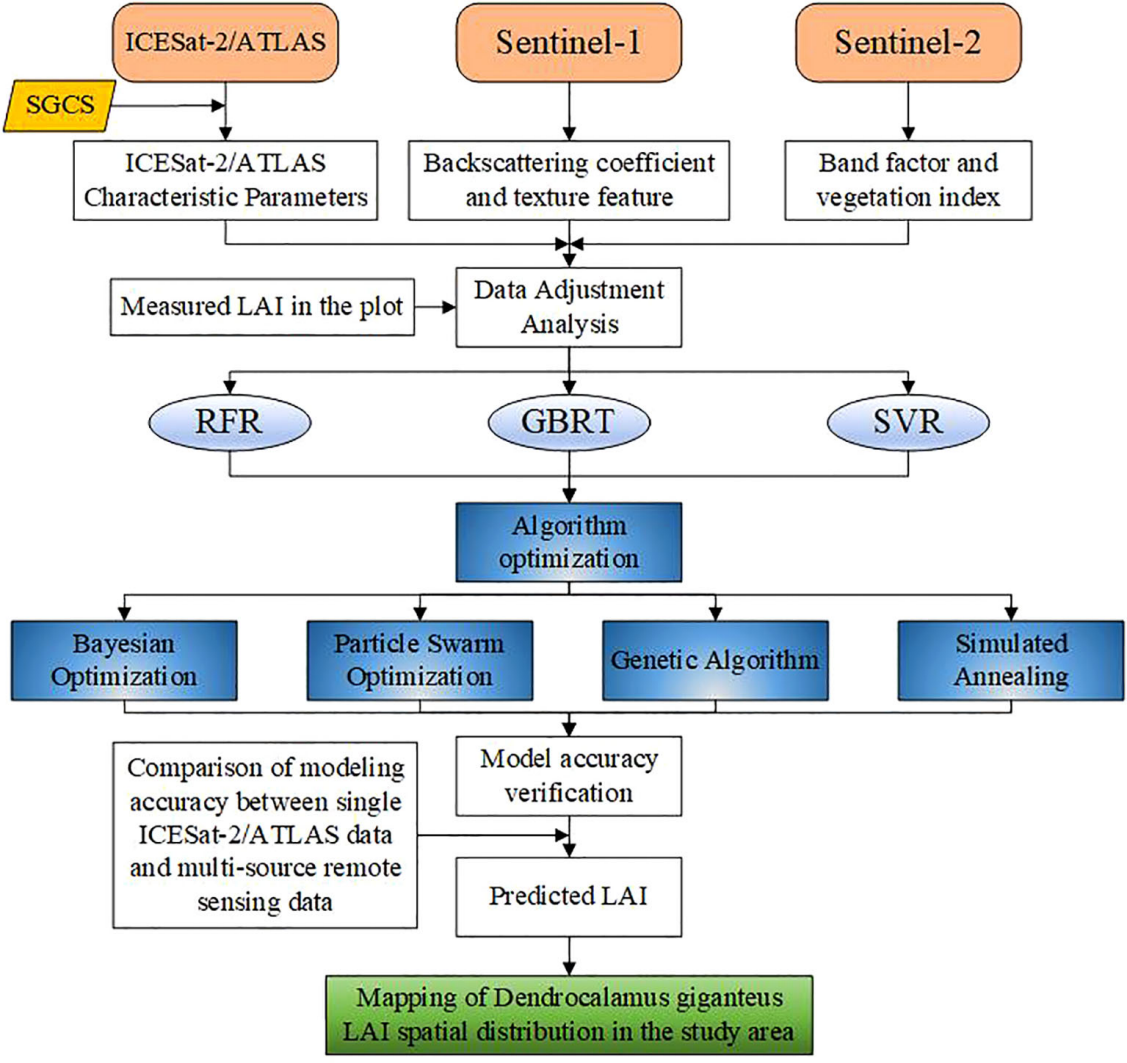
Technology roadmap.

In this research, the SGCS method based on the simple Kriging method was first used to interpolate ICESat-2/ATLAS light spots to obtain planar grid information, and then the ICESat-2/ATLAS variables were coordinated with Sentinel-1/-2 variables. The relationship between variables and measured LAI was assessed using the Pearson correlation coefficient. RFR, GBRT, and SVR were respectively used to construct LAI estimation models. Then, BO, PSO, GA, and SA were used to optimize the RFR, GBRT, and SVR models respectively. Then, the estimation model of LAI was constructed using BO-RFR, BO-GBRT, BO-SVR, PSO-RFR, PSO-GBRT, PSO-SVR, GA-RFR, GA-GBRT, GA-SVR, SA-RFR, SA-GBRT and SA-SVR methods. A spatial distribution map of LAI was generated using the optimal model. **Figure 2** illustrates the technical approach employed in this research.

#### 3.1.1 Geostatistical method

##### 3.1.1.1 Variable handling

To enhance the speed of model convergence, all variables are normalized to adjust their values to fall within the ranges of [0,1] or [-1,1] before performing variance function analysis. Following this step, each variable’s data structure is assessed through a normality test. To better approximate a normal distribution, variables with non-normal distributions are transformed using a cube root function. This transformation ensures the data is appropriately conditioned for the variance function analysis. The following formula is applied:


(1) $$\begin{array}{c} Y=\frac{y-y_{min}}{y_{max}-y_{min}} \end{array}$$

Note: *Y* denotes the outcome of the normalization procedure; while *y* indicates the original value. Additionally, $y_{min}$ is the smallest value within the initial dataset, and $y_{max}$ signifies the largest value in the same dataset.

##### 3.1.1.2 Variogram function

The variogram function, commonly known as the semivariance function, is essential in kriging interpolation and serves as a critical analytical tool in the field of spatial statistics (Wang et al., 2022). The theoretical model’s structural characteristics are defined by four key parameters: the type of function, nugget variance (C_0_), sill (C_0_ + C, with C indicating the partial sill), and range (a). The formula is as follows:


(2) $$\begin{array}{c} \gamma \left(h\right)=\frac{1}{2n\left(h\right)}\sum_{i=1}^{n\left(h\right)}{\left[Z\left(X_{i}+h\right)-Z\left(X_{i}\right)\right]}^{2} \end{array}$$

Note: γ(h) is the variogram, where *h* is the distance between points. n(h) is the number of sample pairs, and $Z\left(X_{i}\right)$ and $Z\left(X_{i}+h\right)$ are the variable values at $X_{i}$ and $X_{i}+h$ .

##### 3.1.1.3 Principle of method

Understanding the spatial complexity of forest ecosystems and accurately estimating their structural parameters rely heavily on geostatistical techniques. Among these, SGCS has emerged as a particularly effective method. Rooted in the Monte Carlo framework, this stochastic simulation technique allows for the generation of multiple equally probable realizations of spatial variables, capturing the inherent variability of vegetation structure across expansive landscapes. Its capacity to account for spatial variability and uncertainty provides a more comprehensive and nuanced understanding of vegetation structure over extensive areas than traditional deterministic methods (Berterretche et al., 2005). Geostatistics is extensively utilized in the field of geosciences to measure the uncertainty associated with regionalized variables (Emery and Peláez, 2011). The Kriging interpolation technique, commonly employed in the field of forestry, is noted for its objectivity and low variance in estimation error. Despite this, the attempt to minimize error variance may inadvertently produce a smoothing effect, which could potentially bias the estimation of overall spatial variability (Zhang et al., 2017). As a result, this technique finds its primary application in localized estimations. Unlike Kriging interpolation, SGCS employs Monte Carlo methods to establish the probability distribution function from the initial dataset. This approach that the variance of simulated values at each location aligns with the original data’s Gaussian distribution, effectively preventing the smoothing effect common to all spatial estimators that aim to minimize mean squared error (Huang et al., 2016; Olea and Pawlowsky, 1996; Qu et al., 2014). SGCS effectively maintains the intensity of spatial variation and produces several equally likely realizations at locations that have not been sampled, thus aiding in the quantification of spatial uncertainty in geographic attributes. Given that the acquired data frequently falls short of meeting the simulation criteria, it is crucial to normalize the data initially, followed by the application of inverse transformation to the simulation outcomes (Luo et al., 2023).

#### 3.1.2 LAI estimation model

##### 3.1.2.1 Random forest regression

RFR is a method that combines several decision trees by utilizing the concept of ensemble learning (Breiman, 2001). RF’s random selection of the training sample makes it insensitive to data noise and is not affected by the collinearity of the predictor (Rhodes et al., 2023). The RFR model in this research was developed using the “randomForest” package in the RStudio (RStudio, version 4.2.2, Posit PBC, Boston, MA, USA, https://rstudio.com/) environment.

##### 3.1.2.2 Gradient boosting regression trees

Unlike Random Forest Regression, GBRT is an integrated method characterized by correction and enhancement (Friedman, 2001), which offers the benefits of high predictive accuracy and rapid processing speed, strong robustness to outliers, and is not easy to fall into overfitting. The GBRT model in this research was executed using the “gbm” package available in RStudio.

##### 3.1.2.3 Support vector machine regression

SVR is a machine learning algorithm commonly applied in supervised learning tasks, extensively utilized in both classification and regression analysis (Jiang et al., 2022). Because SVR focuses on support vectors rather than the entire data set, support vector machines also perform well on small training datasets. The SVR model is implemented utilizing the “e1071” package within the RStudio environment.

#### 3.1.3 LAI estimation model optimization algorithm

##### 3.1.3.1 Bayesian optimization algorithm

**Figure 3:**
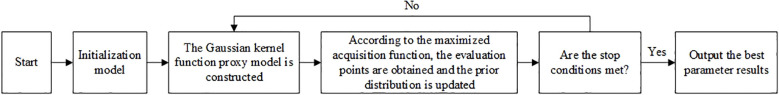
Flow chart of Bayesian optimization algorithm.


BO is an active optimization algorithm proposed by Snoek et al (Snoek et al., 2012). (Wang et al., 2023) in 2012, which is mainly used to solve extreme value problems of functions with unknown expressions. The core of the Bayesian optimization algorithm is composed of two parts: a prior function and a collection function. The prior function calculates the function mean and covariance of each point through Gaussian process regression to obtain a posterior probability. Then, the collection function is constructed by improving the probability of finding the minimum value less than the current function to select the next set of hyperparameters (Shahriari et al., 2015). For the problem of Bayesian optimization hyperparameters, in the hyperparameter search space χ, the Bayesian optimizer optimizes f through a finite number of experiments in an iterative way to get the best combination of hyperparameters. In this study, the “rBayesianOptimization” language package in RStudio was used to realize the Bayesian algorithm optimization of the model, and the algorithm flow is shown in **Figure 3**.

(3) $$\begin{array}{c} x^{*}=\underset{x\in \chi }{\text{argmin}}f\left(x\right) \end{array}$$

Note: $x^{*}$ is the best combination of hyperparameters optimized; f(x) is the target optimization function.

##### 3.1.3.2 Particle swarm optimization algorithm

**Figure 4:**
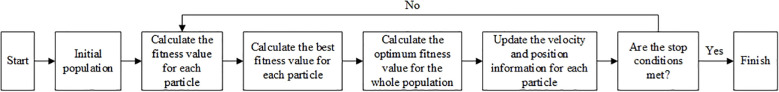
Flow chart of particle swarm optimization algorithm.


PSO, an evolutionary algorithm designed for global optimization, it was first proposed by Kennedy and Eberhart, 1995 (Kennedy and Eberhart, 1995). The PSO algorithm begins by initializing a population of particles, where each particle is assigned an initial position and velocity. The evaluation of the fitness function is subsequently carried out for every particle, and the resulting value is recorded as the best historical solution for that individual. Then, the global historical optimal solution is obtained through cooperation and sharing of information among particles, and the new speed and position are obtained through iteration (Wang et al., 2018). The particle’s fitness function is subsequently reevaluated to assess its updated performance. If the new fitness value exceeds the particle’s previous individual historical optimum, it becomes the new individual best for that particle. Following the update of the optimal solution for each particle’s individual history, the global optimal solution is subsequently revised, and then the iteration continues to obtain the new speed and position (Indrawati and Wahyuni, 2023). In this study, the “pso” language package in RStudio is used to realize the particle swarm optimization of the model, and the algorithm process is shown in **Figure 4**. Utilizing the formula provided, the particle modifies both its velocity and position. Once it determines its individual optimal solution, termed as the individual extremum *P*_best_, in conjunction with the optimal solution identified by the entire population, referred to as the global extremum *g*_best_, the particle then carries out the necessary updates.

(4) $$\begin{array}{c} V^{k+1}=wV^{k}+c_{1}\left(P_{\text{best}}-P\right)rand\left(0,1\right)\\ +c_{2}\left({ɡ}_{\text{best}}-P\right)rand\left(0,1\right) \end{array}$$

(5) $$\begin{array}{c} P^{k+1}=P+V^{k+1} \end{array}$$

Note: *V* denotes the particle velocity, *k* represents the kth iteration, *P* indicates the current position of the particle, rand (0,1) refers to a random number that exists within the bounds of 0 and 1, $c_{1}$ and $c_{2}$ are the learning factors, and *w* is the weighting coefficient.

##### 3.1.3.3 Genetic optimization algorithm

**Figure 5:**
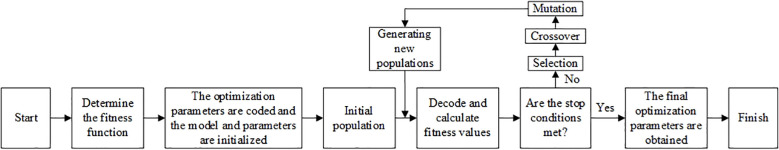
Flow chart of genetic optimization algorithm.

GA were developed through the computational modeling of biological systems, with their initial introduction credited to Holland (Ribeiro-Filho et al., 1994). This approach represents a stochastic technique for global search and optimization that mimics the evolutionary processes found in nature. It is grounded in principles derived from Darwin’s theory of evolution and Mendelian genetics. Essentially, this method operates as a robust parallel search strategy, autonomously collecting and integrating data regarding the search space throughout the exploration phase, while also adapting its search management to pinpoint the optimal solution (Holland, 1992). The primary components of GA include chromosome coding, fitness functions, selection, recombination, and evolution schemes. These components have been successfully applied to address optimization problems involving both continuous functions (differentiable or not) and discrete functions. In this study, the “GA” (Scrucca, 2013) language package in RStudio was used to realize the genetic algorithm optimization of the model, and the algorithm flow is shown in **Figure 5**.

##### 3.1.3.4 Simulated annealing optimization algorithm

**Figure 6:**
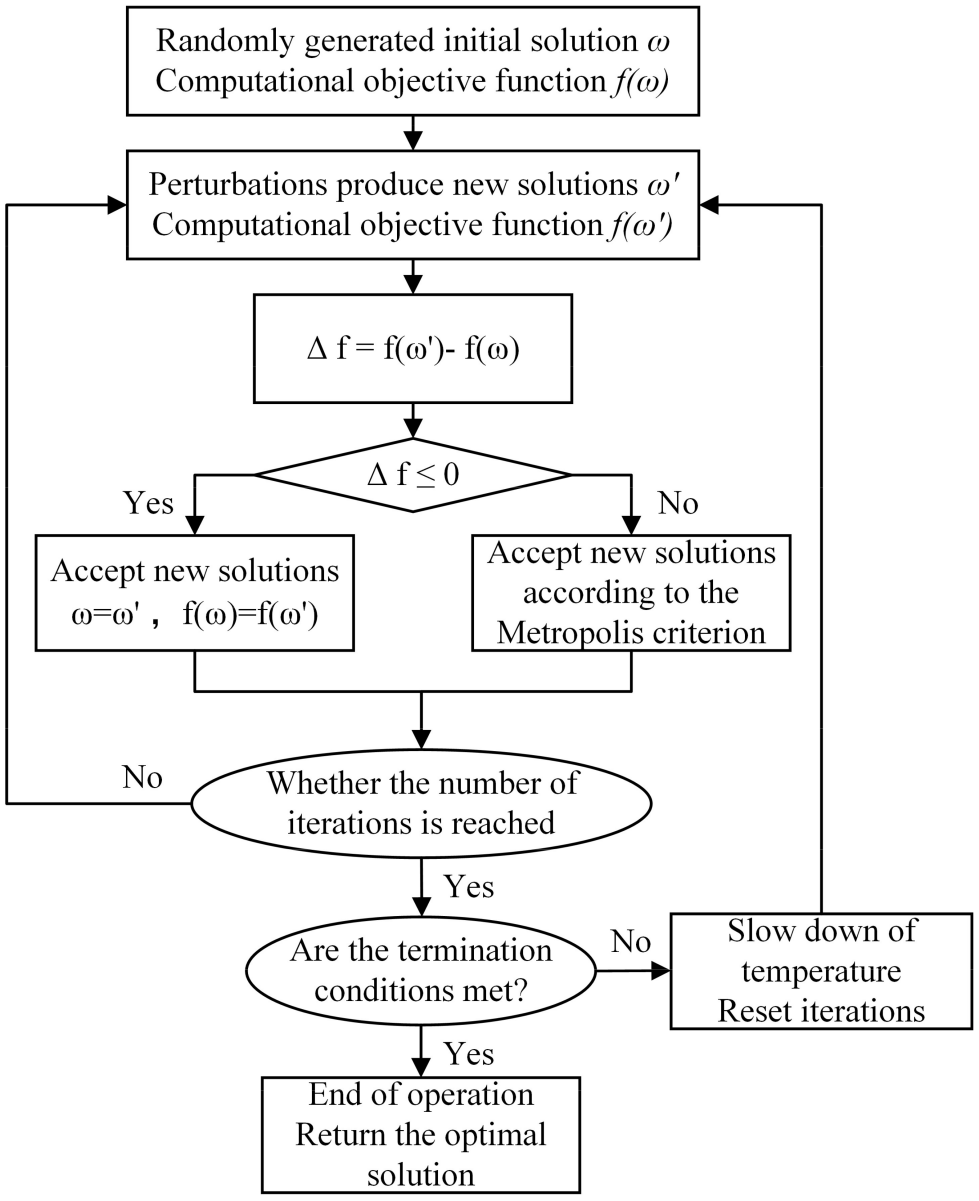
Flow chart of simulated annealing optimization algorithm.

SA algorithm is a heuristic algorithm for solving optimization problems (Rutenbar, 1989). The central concept of the simulated annealing algorithm begins with a high initial temperature that gradually decreases over time. This process incorporates specific probabilistic jump features, allowing the algorithm to explore the solution space randomly in search of the global optimum of the objective function. Consequently, the algorithm is likely to avoid local optima and eventually converge to the global optimum (Suppapitnarm et al., 2000). In this study, the “GenSA” language package in RStudio is used to realize the simulated annealing algorithm optimization of the model, and the algorithm process is shown in **Figure 6**.

##### 3.1.3.5 Model optimization parameters

In this research, we utilized several optimization techniques, including the BO, PSO, GA, and SA, to enhance the RFR, GBRT, and SVR models. The definitions of each optimization parameter for these models are detailed in **Table 1**.

**Table 1:** Description table of parameters for RFR, GBRT and SVR models.

Model type	Parameter	Description
RFR, GBRT	max_depth	Decision tree depth
	n_estimators	Number of decision trees
	min_samples_split	The minimum number of samples required for a node to be divided
	min_samples_leaf	The minimum number of samples that a leaf node contains
SVR	cost_parameter	Regularization parameter
	kernel_coefficient	Kernel parameter of radial basis function

#### 3.1.4 Model accuracy evaluation

In this research, we apply the leave-one-out cross-validation technique to assess the model’s predictive capacity and the precision of its LAI estimations from remote sensing. This approach involves sequentially removing a single data point from the dataset, training the model on the remaining data, and testing it on the omitted point. Repeating this procedure for all data points enables a comprehensive evaluation of the model’s generalization performance and enhances the accuracy of LAI estimation derived from remote sensing (Fushiki, 2011). The evaluation metrics used to assess model performance include the coefficient of determination (*R* ^2^), root mean square error (*RMSE*), mean absolute error (*MAE*), and overall estimation accuracy (*P*_1_). The corresponding formula for the calculation is as follows:


(6) $$R^{2}=1-\frac{\sum_{i=1}^{n}{\left(y_{i}-{\hat{y}}_{i}\right)}^{2}}{\sum_{i=1}^{n}{\left(y_{i}-{\overset{-}{y}}_{i}\right)}^{2}}$$

(7) $$RMSE=\sqrt{\frac{\sum_{i=1}^{n}{\left(y_{i}-{\hat{y}}_{i}\right)}^{2}}{n}}$$

(8) $$\begin{array}{c} MAE=\frac{1}{n}\sum_{i=1}^{n}\left|\left(y_{i}-{\hat{y}}_{i}\right)\right| \end{array}$$

(9) $$\begin{array}{c} P_{1}=\left(1-\frac{RMSE}{\bar{y}}\right)\times 100\% \end{array}$$

In the formula, $y_{i}$ : actual value; ${\hat{y}}_{i}$ : estimated value; $\bar{y}$ : mean of the measured values; *n*: quantity.

### 3.2 Data processing

#### 3.2.1 Data processing of spaceborne LiDAR

##### 3.2.1.1 Photon point cloud denoisings

Photon counting radar is more sensitive in detecting photon signals compared to other laser radars. In the ATLAS system, when acquiring reflected photons from targets such as the ground and canopy, it is often affected by noise photons from solar background and atmospheric scattering (Chen et al., 2019). Therefore, noise removal is an important preprocessing step.

In this study, different densities-based spatial clustering of applications with noise (DDBSCAN) (Zhang and Kerekes, 2014) is used in conjunction with K-nearest neighbors-based (KNNB) combined algorithm de-noises ATLAS photon data. The integrated denoising algorithm has achieved significant improvements in denoising effectiveness. Additionally, in the DDBSCAN algorithm, photon densities are computed across all search directions, with the largest density variation employed as the key metric to minimize the effects of inconsistent photon densities on the algorithm’s performance (Zhang et al., 2021).

##### 3.2.1.2 Photon point cloud classification

To effectively differentiate between ground photons and canopy top photons in the de-noised signal, this study utilizes an enhanced version of the progressive TIN densification filter (PTD) technique for classification purposes (Nie et al., 2017). The recognition accuracy of ground photons by this method is higher in areas with large altitude differences and complex terrain. The algorithm mainly includes four parameters (Nie et al., 2018; Zhu et al., 2018, Zhu et al., 2020a): The predefined window size is set at 200 m. Di represents the distance between unclassified photons and the two closest seed ground photons. At refers to the angle created by the line connecting the unclassified photons to their associated seed ground photons and the line extending to the ground surface. Furthermore, Ds denotes the vertical distance from the unclassified photons to the ground surface (Xi et al., 2023). To begin, choose the photon exhibiting the lowest elevation from each window to function as the initial seed ground photon. Subsequently, connect these seed photons to form the initial segment line, categorizing the remaining photons as unclassified. Once the distance Di for each unclassified photon is calculated, if the maximum Di exceeds the established threshold, photons with elevations below this maximum are extracted from the unclassified set as ground photons, ensuring that no canopy photons are included. To identify the optimal ground segment line, an interval of 10 is established, testing values ranging from 10 to 50 with a threshold of 20. This process involves multiple self-iterations until no new seed ground photons are generated. After the initial section line is generated, the improved section line is generated by the Douglas-Peucker algorithm. A cubic spline interpolation method is employed to fit the ground photons and create a model of the ground surface, enabling the extraction of the final ground photon data. Photons are identified as ground photons only when Ds is below 1 m; the remaining photons are classified as vegetation photons (Zhang et al., 2021).

A total of 21080 light spots in the study area were obtained, and 46 parameter values were extracted using the ICESat-2/ATLAS parameter extraction module constructed by PyCharm IDL (PyCharm, JetBrains, Prague, Czech Republic, https://www.jetbrains.com/) environment.

#### 3.2.2 Synthetic aperture radar and optical image data processing

In this study, the GEE platform was used to download Sentinel-1 data from microwave remote-sensing satellite images and Sentinel-2 data from optical remote-sensing satellite images. The data collection time was from December 2023 to February 2024, and the cloud cover was set at less than 5%. Then the median synthesis and cubic convolution methods were used to resample the remote sensing image data to a resolution of 15×15 m. After resampling, the area is 225 m^2^, allowing it to be matched with the sample area (226.865 m^2^) and the spot area (226.865 m^2^) to minimize the error, which is approximately 0.822%, indicating high precision. Among them, Sentinel-1 data needs to be pre-processed through fine-track correction, multi-view and coherent speckle filtering, geocoding, etc. In addition, the grey level co-occurrence matrix (GLCM) in the second-order texture algorithm is used to generate texture images in ENVI 5.3 software (ENVI, version 5.3, Exelis VIS, Boulder, CO, USA, https://envi.geoscene.cn/). The window size is set to 5×5, step size to 1, gray level to 64, and eight texture feature information is extracted (Holtgrave et al., 2020). Sentinel-2 data need to be pre-processed by radiometric calibration, atmospheric correction, geometric precision correction, topographic radiation correction, etc., and then image feature parameters, are extracted using ENVI 5.3 software.

#### 3.2.3 Selection of feature parameters

##### 3.2.3.1 Spaceborne LiDAR parameters

This research employs the ATL03 and ATL08 data products from the ICESat-2/ATLAS mission. ATL08 data product is a geophysical data product based on ATL03 data after denoising and signal photon classification, which is generated in 100m segments (composed of 5 20m segments) along the direction of the orbit and contains ground elevation information and vegetation height information. The independent variables chosen for modeling in this research, based on ATLAS parameters, are detailed in **Table 2**.

**Table 2:** ICESat-2/ATLAS parameter table.

Serial number	Parameters
1	solar_elevation
2	h_mean_canopy_abs
3	h_te_best_fit
4	h_te_interp

##### 3.2.3.2 Sentinel-1/-2 parameters

This study effectively employed Sentinel-1 imagery to extract backscatter coefficients and texture features. The extracted backscatter coefficients comprise VV (co-polarized) and VH (cross-polarized). The texture features extracted from VV and VH include: Mean, offering an average measure; Variance, signifying the spread of values; Homogeneity, reflecting uniformity; Contrast, highlighting differences between light and dark areas; Dissimilarity, indicating the degree of dissimilarity between pixels; Entropy, measuring randomness or disorder; Second Moment, related to smoothness or roughness; and Correlation, denoting the relationship between neighboring pixels. The calculation formulas for these texture features are clearly presented in **Table 3**.

**Table 3:** Texture feature table (Zhou et al., 2023).

Name	Formula
Mean	$\sum_{i,j=0}^{n-1}iF_{i,j}$
Variance	$\sum_{i,j=0}^{n-1}iF_{i,j}(\text{i},\text{j}-\mu_{i,j}{)}^{2}$
Homogeneity	$\sum_{i,j=0}^{n-1}i\frac{F_{i,j}}{1+{\left(i-j\right)}^{2}}$
Contrast	$\sum_{i,j=0}^{n-1}iF_{i,j}{\left(i-j\right)}^{2}$
Dissimilarity	$\sum_{i,j=0}^{n-1}iF_{i,j}\left|i-j\right|$
Entropy	$\sum_{i,j=0}^{n-1}iF_{i,j}\left|-\ln F_{i,j}\right|$
Second Moment	$\sum_{i,j=0}^{n-1}iF_{i,j}^{2}$
Correlation	$\sum_{i,j=0}^{n-1}F_{i,j}\left[\frac{\left(i-\mu_{i}\right)\left(j-\mu_{j}\right)}{\sqrt{VA_{i}VA_{j}}}\right]$

Additionally, by utilizing Sentinel-2 imagery, the study extracted original single-band factors and a diverse array of vegetation indices. The extracted original single-band factors encompass bands 2, 3, 4, 5, 6, 7, 8, and 8A. The vegetation indices consist of the Normalized Difference Vegetation Index (NDVI), widely used for assessing vegetation health; Difference Vegetation Index (DVI), providing another measure of vegetation difference; Soil-Adjusted Vegetation Index (SAVI), accounting for soil effects; Optimized Soil-Adjusted Vegetation Index (OSAVI), an improved version of SAVI; Enhanced Vegetation Index (EVI), enhancing vegetation detection; Two-band Enhanced Vegetation Index (EVI2), a variation of EVI; Ratio Vegetation Index (RVI), based on a ratio of bands; Modified Soil-Adjusted Vegetation Index (MSAVI), a modified form of SAVI; Green Normalized Difference Vegetation Index (GNDVI), focusing on green vegetation; Green Ratio Vegetation Index (GRVI), related to green vegetation ratio; Renormalized Difference Vegetation Index (RDVI), and Infrared Difference Vegetation Index (IDVI). The calculation formulas for these vegetation indices are meticulously shown in **Table 4**.

**Table 4:** Calculation formula table of vegetation index factor.

Name	Formula
NDVI (Rouse, 1973)	$\frac{\text{B}8-\text{B}4}{\text{B}8+\text{B}4}$
DVI (Clevers, 1986)	B8−B4
SAVI (Huete, 1988)	$\frac{\left(\text{B}8-\text{B}4\right)\left(1+L\right)}{\text{B}8+\text{B}4+L}$
OSAVI (Rondeaux et al., 1996)	$\frac{\text{B}8-\text{B}4}{\text{B}8+\text{B}4+0.16}$
EVI (Huete et al., 2002)	$2.5\times \frac{\text{B}8-\text{B}4}{\text{B}8+6\text{B}4-7.5\text{B}2+1}$
EVI2 (Jiang et al., 2008)	$2.5\frac{\text{B}8-\text{B}4}{\text{B}8+\text{B}4+1}$
RVI (Pearson and Miller, 1972)	$\frac{\text{B}8}{\text{B}4}$
MSAVI (Qi et al., 1994)	$\frac{2\text{B}8+1-\sqrt{{\left(2\text{B}8+1\right)}^{2}-8\left(\text{B}8-\text{B}4\right)}}{2}$
GNDVI (Gitelson and Merzlyak, 1998)	$\frac{\text{B}8-\text{B}3}{\text{B}8+\text{B}3}$
GRVI (Rouse et al., 1974b)	$\frac{\text{B}3}{\text{B}4}$
RDVI (Rouse et al., 1974b)	$\frac{\text{B}8-\text{B}4}{\sqrt{\text{B}8+\text{B}4}}$
IDVI (Rouse et al., 1974a)	$\frac{\text{B}8-\text{B}3}{\text{B}8+\text{B}3}$

## 4 Results and analysis

### 4.1 Sequential gaussian condition simulation effect

#### 4.1.1 Selection of the variogram model

**Figure 7:**
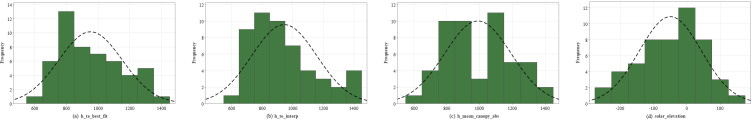
Normal distribution plot of characteristic variables. **(A)** h_te_best_fit; **(B)** h_te_interp; **(C)** h_mean_canopy_abs; **(D)** solar_elevation.

Before fitting the variance function model, testing the normal distribution of ATLAS feature variables is essential. As shown in **Figure 7**, the four feature variables are basically in line with normal distribution. Then, the variance function is fitted using models within the GS+9.0 software (GS+, version 9.0, Gamma Design Software, LLC, Plainwell, MI, USA, https://gs.software.informer.com/), and the best theoretical model is selected from them. The variance function analysis results are presented in **Table 5**. The findings indicate that: (1) The models with the best effects of the four parameters are all spherical models. (2) All the four parameters have medium-high spatial autocorrelation.

**Table 5:** Variation function analysis table.

Parameters	Model	*R* ^2^	RSS	C_0_	C_0_+C	C_0_/C_0_+C	Range/m
h_te_best_fit	Spherical	0.921	0.567	0.051	2.397	0.979	27500
	Exponential	0.87	1	0.001	2.465	1.000	33300
	Gaussian	0.914	0.618	0.314	2.392	0.869	22516.66
h_te_interp	Spherical	0.921	0.567	0.052	2.398	0.978	27600
	Exponential	0.87	1	0.001	2.465	1.000	33300
	Gaussian	0.914	0.618	0.319	2.392	0.867	22516.66
h_mean_canopy_abs	Spherical	0.925	0.406	0.163	2.197	0.926	27800
	Exponential	0.877	0.687	0.001	2.249	1.000	31800
	Gaussian	0.917	0.447	0.394	2.192	0.820	22689.87
solar_elevation	Spherical	0.74	4.241	710	2151	0.670	13000
	Exponential	0.69	5.043	259	2152	0.880	11700
	Gaussian	0.732	4.359	947	2151	0.560	11085.13

#### 4.1.2 Interpolation effect

**Figure 8:**
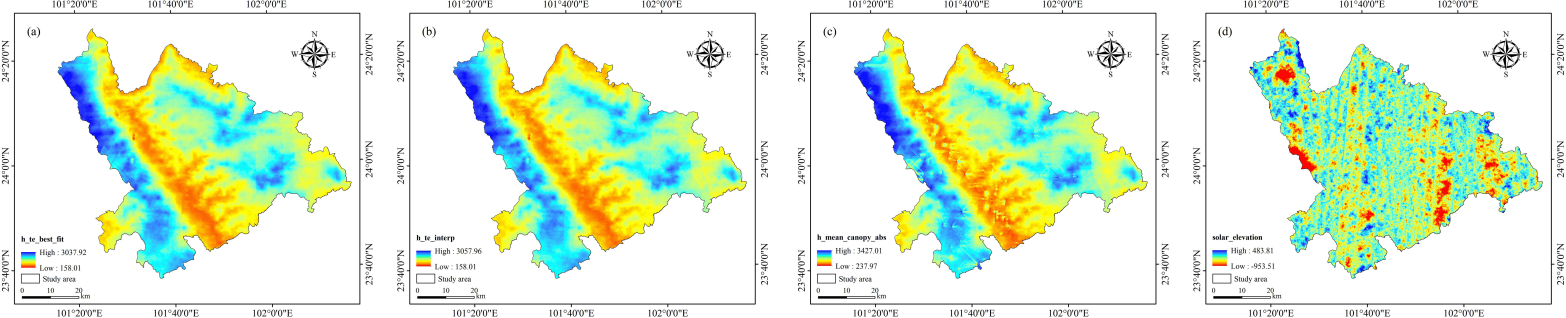
Spatial interpolation renderings of characteristic variables. **(A)** h_te_best_fit; **(B)** h_te_interp; **(C)** h_mean_canopy_abs; **(D)** solar_elevation.

In this research, GS+9.0 software and ArcGIS10.8 software (ArcGIS, version 10.8, ESRI, Redlands, CA, USA, https://www.esri.com/) were used to analyze the SGCS of LAI. First, GS+9.0 software was used for variance function analysis, and ArcGIS10.8 software was used for simple Kriging interpolation with the variable range and partial base value of the optimal variance function model, and then SGCS analysis was carried out with the Gauss statistical simulation tool. The study performed simulations at 1, 10, 25, 50, 75, and 100 iterations to determine the optimal number required for robust outcomes (Luo et al., 2023). The findings from the experiments indicate that as the number of SGCS simulations rises, there is a marked increase in the pixel-level variance of the LAI for *Dendrocalamus giganteus*. When the SGCS simulations total 25 iterations, signs of stabilization in the coefficient of variation become evident. Consequently, this study has determined that the optimal threshold for simulations should be set at 25. The effects of SGCS are illustrated in **Figure 8**.

**Figure 9:**
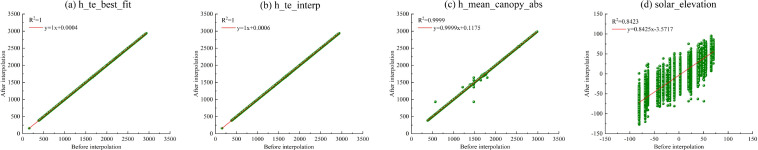
Accuracy verification diagram of spatial interpolation for characteristic variables. **(A)** h_te_best_fit; **(B)** h_te_interp; **(C)** h_mean_canopy_abs; **(D)** solar_elevation.

Spatial interpolation is performed through SGCS to obtain the surface data of the study area. Since the accuracy of spatial interpolation will affect the quality of ICESat-2/ATLAS data and may cause errors to propagate into the prediction model constructed subsequently, we use the 21080 light spots before interpolation and the 21080 light spots after interpolation to perform linear regression analysis and use *R* ^2^ as the evaluation index to verify the accuracy of spatial interpolation. The effect is shown in **Figure 9**.

As shown in the figure above, the *R* ^2^ of the four parameters are 1, 1, 0.9999, and 0.8423 respectively. Three of the parameters have extremely high spatial interpolation accuracy, and one parameter has a relatively high spatial interpolation accuracy, indicating that they can all be used as remote sensing estimation model parameters.

### 4.2 Variables correlation analysis

**Figure 10:**
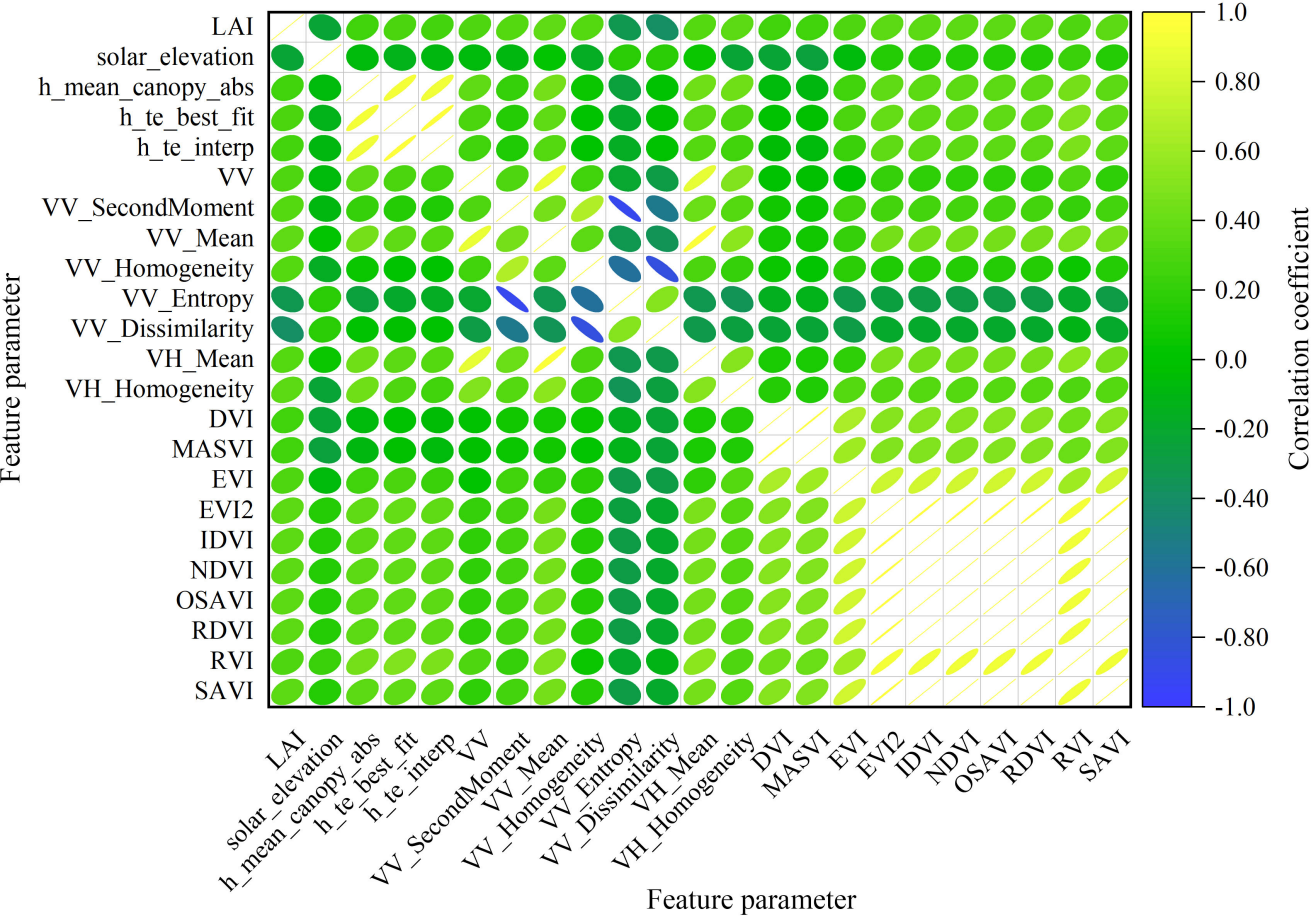
Correlation coefficient thermal matrix diagram.

A total of 84 model feature variables were selected in this study, including 46 ICESat-2/ATLAS feature parameters, 2 Sentinel-1 backscattering coefficients, 16 Sentinel-1 texture feature factors, 8 Sentinel-2 band factors, and 12 Sentinel-2 vegetation index factors. Through an analysis utilizing the Pearson correlation coefficient, parameters demonstrating significant correlations with LAI were identified as independent variables for the model at significance levels of 0.01, 0.05, and 0.1. The parameter matrix with significant correlation is shown in **Figure 10**. At the 0.01 significance level, the significant variables include VV_Mean and VV_Dissimilarity, with Pearson correlation coefficients of 0.367 and 0.384, respectively. At the 0.05 significance level, the significant variables are h_te_best_fit, VV, VV_SecondMoment, VV_Homogeneity, VV_Entropy, VH_Mean, VH_Homogeneity, EVI, EVI2, IDVI, NDVI, OSAVI, RDVI, RVI, and SAVI, with Pearson correlation coefficients of 0.298, 0.315, 0.336, 0.324, -0.336, 0.329, 0.352, 0.319, 0.343, 0.341, 0.341, 0.341, 0.341, 0.31, and 0.341, respectively. At the 0.1 significance level, the significant variables include h_te_interp, solar_elevation, h_mean_canopy_abs, 0.252, and 0.243, with Pearson correlation coefficients of 0.271, -0.236, 0.248, 0.252, and 0.243, respectively.

### 4.3 Prediction model

In this study, spaceborne LiDAR ICESat-2/ATLAS, synthetic aperture radar Sentinel-1, and optical remote sensing image Sentinel-2 were used as data sources to extract feature factors, and Pearson correlation analysis was used to screen out model modeling factors. The modeling factors selected from the ICESat-2/ATLAS dataset include h_te_best_fit, h_te_interp, h_mean_canopy_abs, and solar_elevation. For Sentinel-1, the chosen factors are VV_Mean and VV_Dissimilarity. In the case of Sentinel-2, the selected modeling factors are EVI2 and NDVI. RFR, GBRT, SVR, BO-RFR, BO-GBRT, BO-SVR, PSO-RFR, PSO-GBRT, PSO-SVR, GA-RFR, GA-GBRT, GA-SVR, SA-RFR, SA-GBRT, SA-SVR were used to model the measured LAI and *R* ^2^, *RMSE*, *MAE* and *P*_1_ were used to evaluate the accuracy of the model.

#### 4.3.1 Establishment of the model based on a single ICESat-2/ATLAS data

##### 4.3.1.1 Estimate results based on unoptimized models

**Figure 11:**
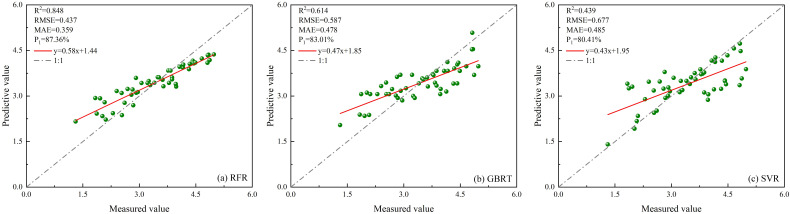
Scatter plots of three unoptimized models constructed using only single ICESat-2/ATLAS data. **(A)** RFR; **(B)** GBRT; **(C)** SVR.

Four feature parameters of ICESat-2/ATLAS data were selected as modeling indices for the RFR, GBRT, and SVR models, and the results are shown in **Figure 11**.

##### 4.3.1.2 Estimation results based on optimization model

**Figure 12:**
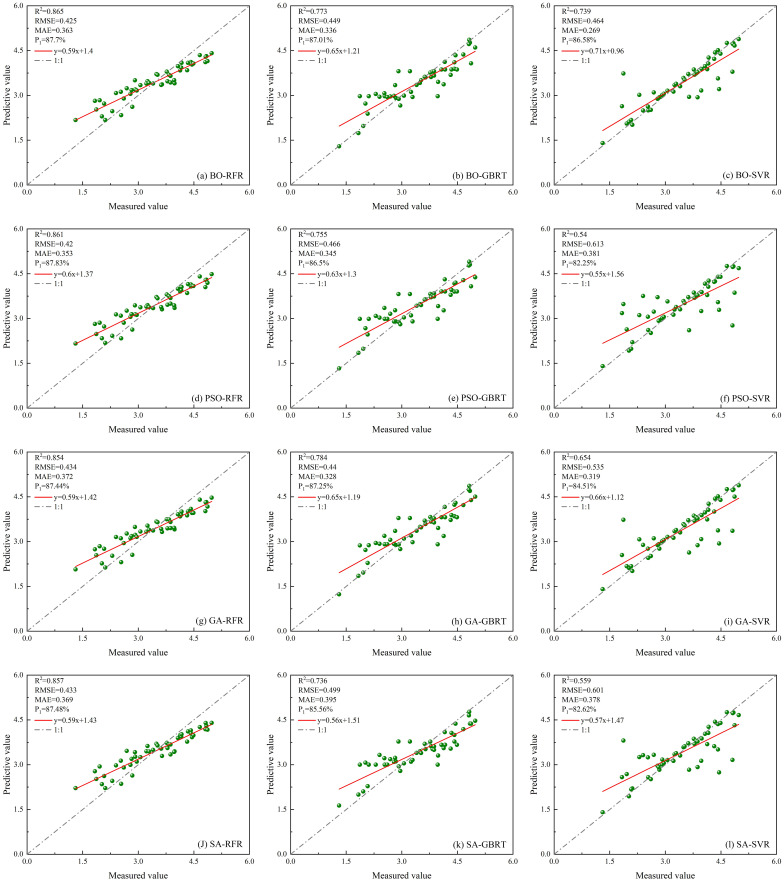
Scatter plots of twelve optimized models constructed using only single ICESat-2/ATLAS data. **(A)** BO-RFR; **(B)** BO-GBRT; **(C)** BO-SVR; **(D)** PSO-RFR; **(E)** PSO-GBRT; **(F)** PSO-SVR; **(G)** GA-RFR; **(H)** GA-GBRT; **(I)** GA-SVR; **(J)** SA-RFR; **(K)** SA-GBRT; **(L)** SA-SVR.

A total of four characteristic parameters of ICESat-2/ATLAS data are selected as BO-RFR, BO-GBRT, BO-SVR, PSO-RFR, PSO-2GBRT, PSO- SVR, GA-RFR, GA-GBRT, GA-SVR, SA-RFR, SA-GBRT, SA-SVR model modeling metrics, and the results are shown in **Figure 12**.

#### 4.3.2 Establishment of model based on multi-source remote sensing data

##### 4.3.2.1 Estimate results based on unoptimized models

**Figure 13:**
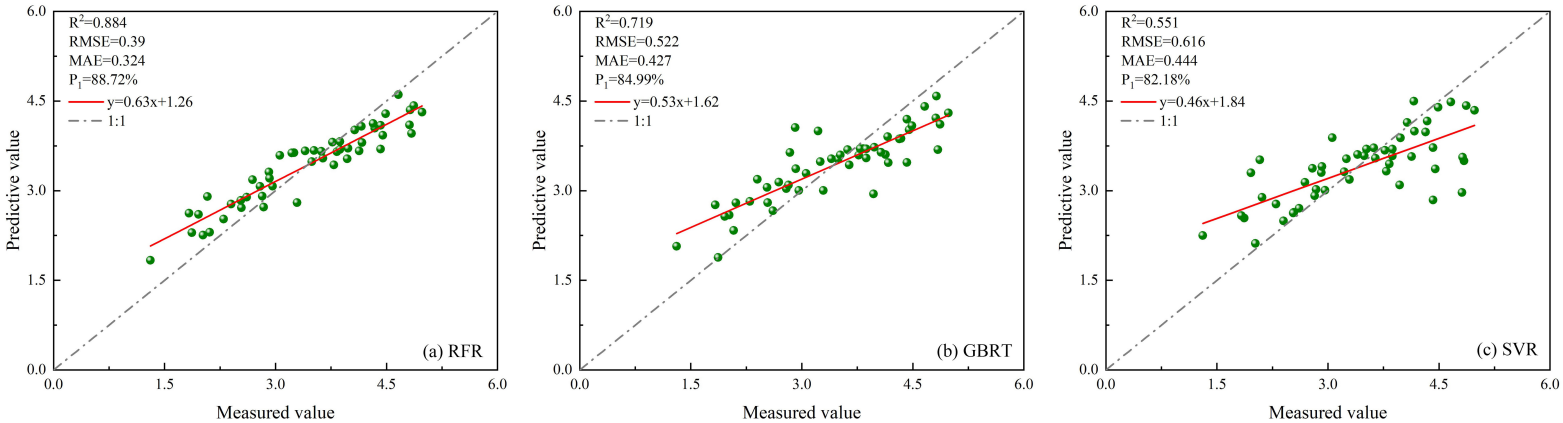
Scatter plots of three unoptimized models constructed using multi-source remote sensing data. **(A)** RFR; **(B)** GBRT; **(C)** SVR.

A total of eight characteristic parameters from ICESat-2/ATLAS data and Sentinel-1/-2 data were selected as modeling factors for RFR, GBRT, and SVR models. The results are shown in **Figure 13**.

##### 4.3.2.2 Estimation results based on optimization model

**Figure 14:**
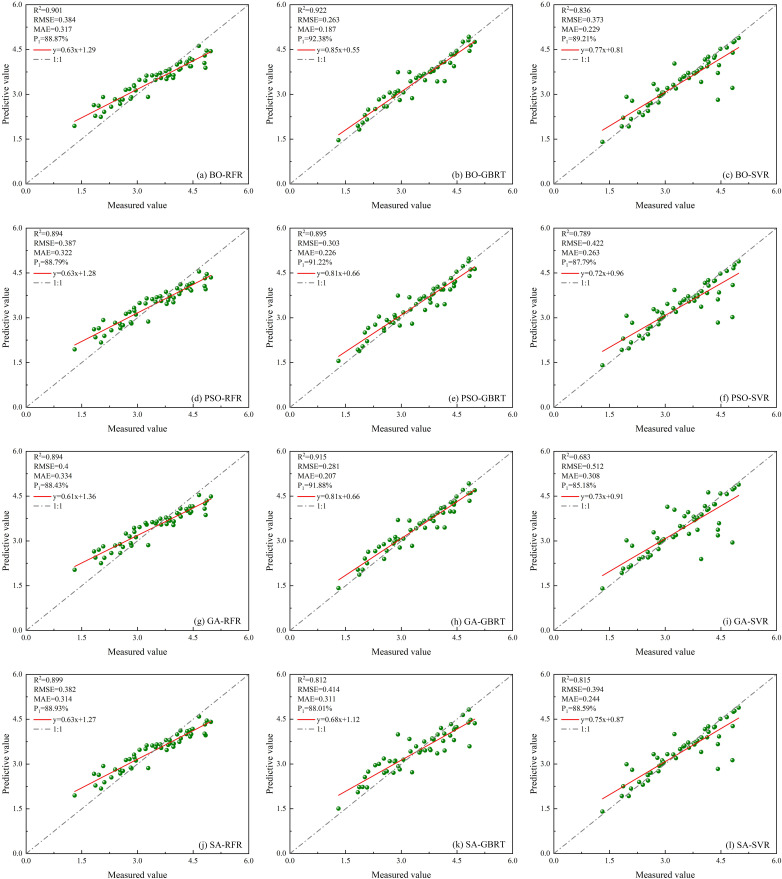
Scatter plots of twelve optimized models constructed using multi-source remote sensing data. **(A)** BO-RFR; **(B)** BO-GBRT; **(C)** BO-SVR; **(D)** PSO-RFR; **(E)** PSO-GBRT; **(F)** PSO-SVR; **(G)** GA-RFR; **(H)** GA-GBRT; **(I)** GA-SVR; **(J)** SA-RFR; **(K)** SA-GBRT; **(L)** SA-SVR.

Select eight characteristic parameters from ICESat-2/ATLAS data and Sentinel-1/-2 data as modeling factors for BO-RFR, BO-GBRT, BO-SVR, PSO-RFR, PSO-GBRT, PSO-SVR, GA-RFR, GA-GBRT, GA-SVR, SA-RFR, SA-GBRT, and SA-SVR models. The result is shown in **Figure 14**.

### 4.4 Comparison of model effects

**Figure 15:**
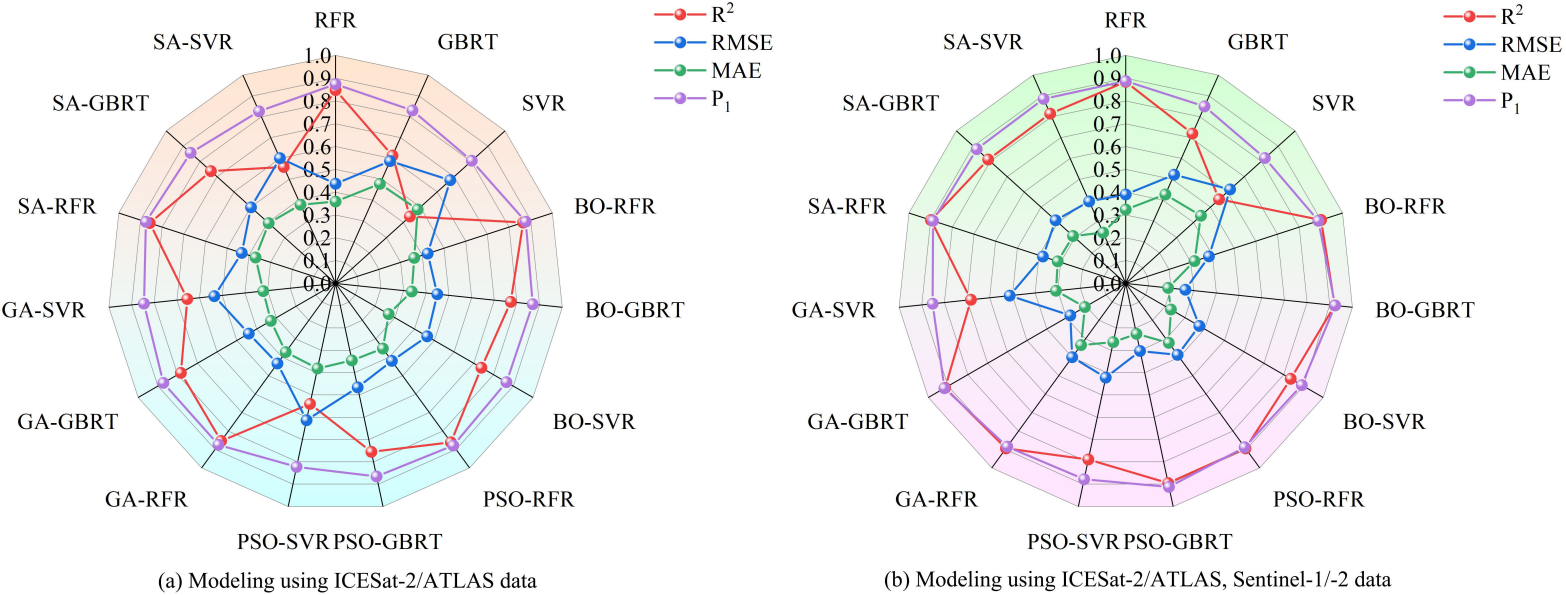
Radar charts used for comparing the accuracy of models. **(A)** Modeling using ICESat-2/ATLAS data; **(B)** Modeling using ICESat-2/ATLAS, Sentinel-1/-2 data.

Based on ICESat-2/ATLAS satellite-borne LiDAR data and combining multiple remote sensing data, the study establishes LAI estimation models for single remote sensing data as well as multi-source remote sensing data using RFR, GBRT, and SVR models optimized by BO, PSO, GA, and SA, respectively, and the radar effect diagram generated by the combination of all models is shown in **Figure 15**. According to the model effect, the accuracy of RFR, GBRT, and SVR models optimized by the four optimization algorithms has been improved to varying degrees compared with that before optimization. The BO-GBRT model constructed by combining ICESat-2/ATLAS, and Sentinel-1/-2 data has the best effect. *R* ^2^, *RMSE*, *MAE*, and *P*_1_ were 0.922, 0.263, 0.187, and 92.38%, respectively. Compared with the constructed GBRT model, *R* ^2^ increased by 20.3%, *RMSE* decreased by 25.9%, *MAE* decreased by 24% and *P*_1_ increased by 7.39%. Among the RFR optimization models constructed with single ICESat-2/ATLAS data, the BO-RFR model had the best effect (*R* ^2^ = 0.865, *RMSE*=0.425, *MAE*=0.363, *P*_1_ = 87.70%). Among the GBRT optimization models, the GA-GBRT model had the best effect (*R* ^2^ = 0.784, *RMSE*=0.44, *MAE*=0.328, *P*_1_ = 87.25%). Among the SVR optimization models, the BO-SVR model has the best effect (*R* ^2^ = 0.739, *RMSE*=0.464, *MAE*=0.269, *P*_1_ = 86.58%). The optimization model with the worst effect was PSO-SVR (*R* ^2^ = 0.54, *RMSE*=0.613, *MAE*=0.381, *P*_1_ = 82.25%). In the RFR optimization model constructed by multi-source remote sensing data, the BO-RFR model has the best effect (*R* ^2^ = 0.901, *RMSE*=0.384, *MAE*=0.317, *P*_1_ = 88.87%). Among the GBRT optimization models, the BO-GBRT model had the best effect (*R* ^2^ = 0.922, *RMSE*=0.263, *MAE*=0.187, *P*_1_ = 92.38%). Among the SVR optimization models, the BO-SVR model has the best effect (*R* ^2^ = 0.836, *RMSE*=0.373, *MAE*=0.229, *P*_1_ = 89.21%). The least effective optimization model was GA-SVR (*R* ^2^ = 0.683, *RMSE*=0.512, *MAE*=0.308, *P*_1_ = 85.18%). When the model remained unchanged and the data sources used were changed, the accuracy of the LAI remote sensing estimation model constructed with multi-source remote sensing data was superior to that of the LAI remote sensing estimation model constructed with single ICESat-2/ATLAS data. Research indicates that integrating ICESat-2/ATLAS data with additional remote sensing datasets, and employing multi-source remote sensing methods to estimate LAI, significantly enhances the accuracy of LAI estimations. Moreover, various optimization algorithms are used to optimize machine learning methods, which can further improve the accuracy of LAI estimation.

### 4.5 Spatial distribution of LAI

**Figure 16:**
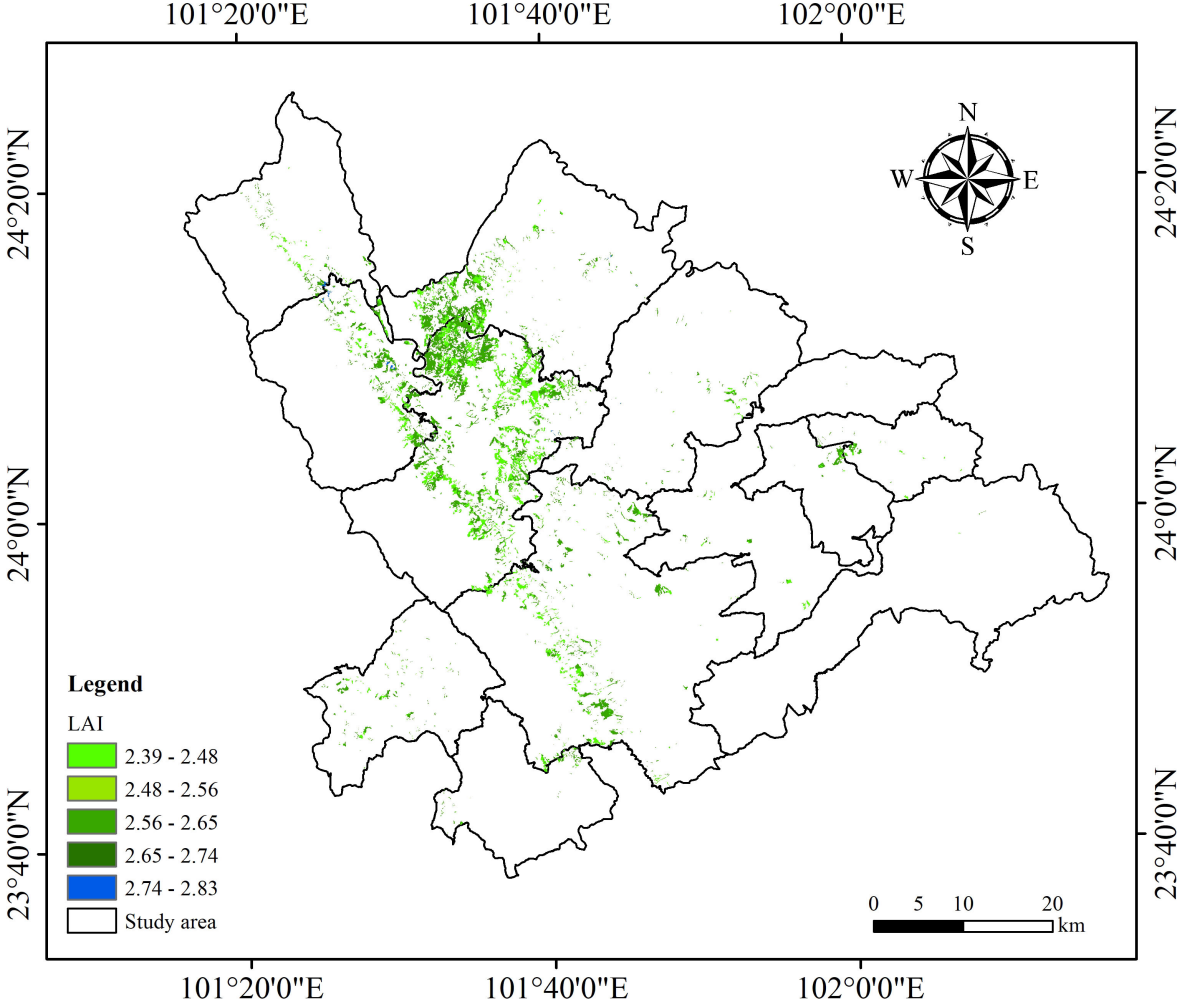
The spatial distribution map of LAI in the study area.

This study utilized data from ICESat-2/ATLAS, Sentinel-1/-2, applying SGCS combined with an optimized model to estimate the spatial distribution of LAI for *Dendrocalamus giganteus* in Xinping County. To improve the accuracy of RFR, GBRT, and SVR models, the study incorporated BO, PSO, GA, and SA. When comparing machine learning models based on single-source ICESat-2/ATLAS data with those integrating multi-source remote sensing data, it was found that the BO-GBRT model, which used the combined data, achieved the highest performance. **Figure 16** illustrates the spatial variation of LAI across the study area. The map reveals pronounced differences in the distribution of *Dendrocalamus giganteus* LAI within Xinping County, mainly ranging from 2.39 to 2.83, with an average value of 2.61. The high-value LAI areas were mainly near Garsha Town, Laochang Town, and Shuitang Town, while the low-value LAI areas were scattered and diversified. Due to the influence of local climatic conditions, soil types, and other environmental factors, these regions do not have obvious regular distribution.

## 5 Discussion

### 5.1 Feature variables choice

**Figure 17:**
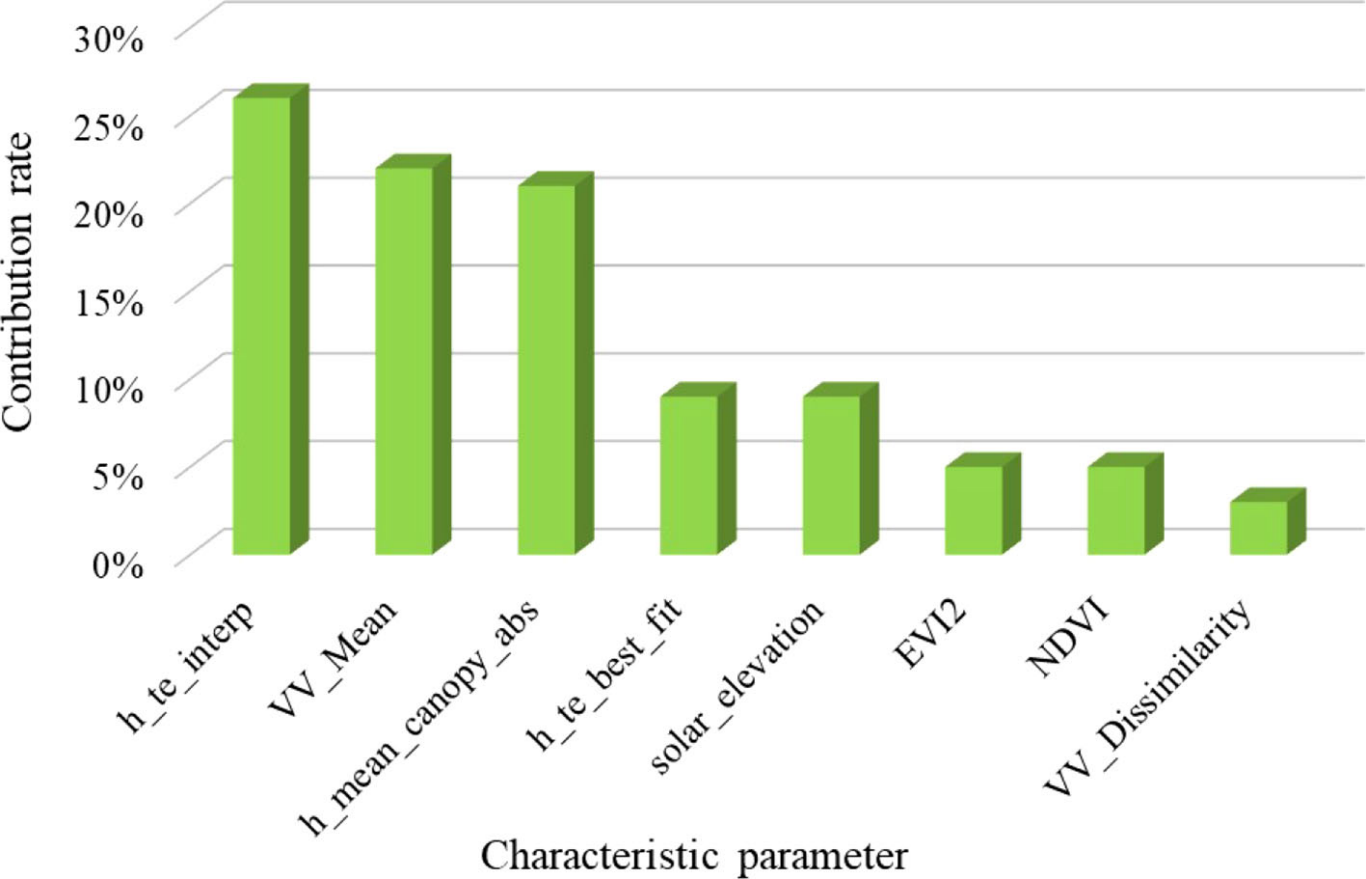
The contribution rate chart of modeling factors.

The selection and combination of characteristic variable factors largely determine the accuracy of the prediction model and inversion results (Zhang et al., 2022). This study optimized the parameters of ICESat-2/ATLAS through an analysis using the Pearson correlation coefficient, and the four variables with the highest correlation coefficients were obtained as the dependent variables of the LAI estimation model. In the selection of regional scale feature variables, optical remote sensing data is susceptible to different degrees of “light saturation” effect of forest vegetation. Single-band reflectance is the most significantly impacted, with the vegetation index experiencing effects to a slightly lesser extent, and texture features can represent the structure information of ground objects in remote sensing images and reflect the spatial change of land cover type (Shu et al., 2022; Zhao et al., 2016), and are the least affected by light saturation. In this paper, the contribution rate of parameters used by each data source in the remote sensing estimation model is shown in **Figure 17**. The contribution rate is 26%, 22%, 21%, 9%, 9%, 5%, 5%, and 3% in descending order. The contribution rate of spaceborne Lidar data is the highest, followed by SAR data, and the contribution rate of optical remote sensing data is the lowest, the SAR factor is added to realize the integration of multi-sensor data to solve the light saturation problem of optical image data, to enhance the precision of the model’s estimates. The intricate nature of the terrain significantly affects the quality of optical and microwave remote sensing images. Moreover, spaceborne LiDAR footprints often exhibit a sparse and uneven spatial distribution, which complicates precise measurements (Zhao et al., 2024). These challenges, when combined, introduce considerable uncertainty in estimating the LAI if only one type of remote sensing data is utilized. Consequently, depending solely on a single data source for LAI estimation can result in substantial inaccuracies, especially in environments characterized by diverse topography. As a result, researchers often merge data from various remote sensing sources to enhance the precision of LAI estimations, thereby addressing the limitations inherent in relying solely on a single data set.

### 5.2 Effect of spatial interpolation

The spatial interpolation techniques in geostatistics demand specific conditions regarding the quantity and spatial distribution of data points. The primary factors that constrain the precision of geostatistical estimates are the correctness of data point parameters, the selected interpolation technique along with the attributes of the employed interpolation model. Spatial interpolation technology can be used to predict the values of unknown regions based on the measured data of known regions. This method can make full use of the dense light spots of spaceborne LiDAR to achieve large-scale forest LAI inversion. For example, Narine et al (Narine et al., 2020). use ICESat-2 and Landsat 8 data. The AGB inversion results are extrapolated to the whole study area, so as to realize the estimation of AGB at the regional scale. The distribution of the footprint from spaceborne LiDAR is generally uniform along the ground track instead of being random, forest LAI obtained by interpolating ICESat-2/ATLAS data may show a significant smoothing effect. To address this issue and ensure the acquisition of high-quality data, this study first adopts the simple Kriging interpolation method, and then uses the Gauss statistical simulation tool for SGCS analysis, thus effectively reducing the smoothing effect in the process of spatial interpolation. Xu et al (Xu et al., 2023). removed low-quality light spots from the strip prior to applying interpolation with the satellite-borne LiDAR data. This process involved discarding certain neighboring strips and points within the same strip, which helped to minimize the smoothing effect during the subsequent interpolation. Furthermore, because the spot tracks of ICESat-2 and GEDI differ, the spots from GEDI can effectively disrupt the uniform distribution of the ICESat-2 spots, thereby reducing the smoothing effect in the interpolation process and obtaining high-precision LAI estimation results. Liu et al (Liu et al., 2022), for example, developed a novel neural network guided interpolation (NNGI) method. The forest canopy height distribution map was created through the integration of data from GEDI, ICESat-2/ATLAS, and Sentinel-2. A comparative analysis was conducted using the GEDI verification footprint, UAV LiDAR data, and field measurements. The results showed that the *R* ^2^ values and *RMSE* for canopy height obtained via NNGI interpolation fell between 0.55 and 0.60, with *RMSE* values ranging from 4.88 to 5.32 m. These findings suggest that the NNGI method has effectively derived the 30 m resolution forest canopy height distribution across China. In this paper, GS+9 software was used to conduct variance function analysis, and the optimal model of each ATLAS parameter interpolation was determined. Compared with the default interpolation model, the optimal model selected based on variance function obtained higher accuracy of interpolation results. In addition, the adoption of the SGCS method effectively reduced the appearance of the smoothing effect. The practicality of applying spatial interpolation techniques to extrapolate point data across the entire area has been demonstrated.

### 5.3 Model optimization and selection

In this study, there is an error transfer between the spatial interpolation method and the machine learning model in the process of LiDAR spot scale conversion. The accuracy of the prediction model is significantly influenced by factors such as the sample size of the measured data, the uncertainty inherent in the remote sensing model, and the variability of its parameters. When the quantity of measured data increases, the predictive model becomes more representative, and the associated uncertainty decreases. However, once the sample size reaches a specific threshold, further increases in the number of samples no longer lead to substantial improvements in the model’s accuracy. In this research, 51 LAI data points were collected, with sampling locations widely distributed to adhere to the principle of maximizing sample diversity and to meet the precision standards required for field investigations (Hua and Zhao, 2021; Song et al., 2022). To further minimize the uncertainty in the prediction outcomes, four optimization algorithms, BO, PSO, GA, and SA, were selected to optimize the performance of RFR, GBRT, and SVR models respectively. The results show that using the optimization algorithm can significantly enhances the prediction accuracy of the machine learning model, and the BO-GBRT model constructed by collaborating with multi-source remote sensing data has the best effect (*R* ^2^ = 0.922, *RMSE*=0.263, *MAE*=0.187, and *P*_1_ = 92.38%), and comparing with the GBRT model before the optimization, the *R* ^2^ is improved by 20.3%, the *RMSE* is reduced by 25.9%, and the 24% reduction in *MAE*, and 7.39% improvement in *P*_1_. Through continuous iterative improvement of the original model, the BO-GBRT model makes each new model produce smaller errors than the last model and builds a new combined model in the gradient direction of reduced residual (Breiman, 2001). Compared with PSO, GA, and SA algorithms, the BO algorithm can achieve a higher model operation rate and model estimation accuracy with fewer optimization times (Cho et al., 2020). Only four optimization algorithms are tried in this study, and other optimization algorithms or deep forest algorithms can be added in the later stage so that small sample data can also be studied by deep neural network learning and fitting (Xia et al., 2022; Zhou and Feng, 2019). Moreover, this paper does not study the differentiation of strong and weak beams of ICESat-2/ATLAS. Existing studies show that, when forest height estimation is performed, the weak beam exhibits inferior performance compared to the strong beam, and the daytime data is worse than that of the night data (Zhu et al., 2020b). In the future, the influence of the strong and weak beams of ICESat-2/ATLAS on the estimation accuracy of LAI can be further explored.

## 6 Conclusion

In this research, we developed an innovative approach for estimating large-scale LAI by combining data from multiple remote sensing sources with an optimization framework. The strength of ICESat-2 lies in its capability to penetrate tree canopies and deliver high-precision forest structure information. Sentinel-1 can penetrate clouds, rain, and fog, enabling all-weather and all-time observations. Sentinel-2 offers high-resolution vegetation monitoring data. This method leverages the complementary strengths of these three sensors to enhance the reliability of data sources. Additionally, using four optimization algorithms—namely BO, PSO, GA, and SA—we optimized the RFR, GBRT, and SVR models, significantly enhancing the LAI inversion accuracy. Among the 30 models created, which utilized either individual ICESat-2/ATLAS data or a combination of various remote sensing datasets, the BO-GBRT model, which integrates data from ICESat-2/ATLAS along with Sentinel-1/-2, demonstrated the most accurate results. The *R* ^2^, *RMSE*, *MAE*, and *P*_1_ values are 0.922, 0.263, 0.187, and 92.38%, respectively. Compared to the GBRT model constructed using the same data source, the *R* ^2^ increased by 20.3%, the *RMSE* decreased by 25.9%, the *MAE* decreased by 24%, and the *P*_1_ increased by 7.39%. This demonstrates a significant optimization effect. Therefore, this method proves to be efficient and accurate for large-scale LAI estimation. Compared to estimation accuracy using a single data source, our method provides more reliable results. In comparison to existing traditional machine learning methods, our approach demonstrates superior performance. However, this method still exhibits deviations when estimating areas with high vegetation density or extensive occlusions. In future research, incorporating additional environmental factors or deep learning methods could further refine our approach, achieving larger-scale and higher-precision estimation of vegetation structure parameters.

## Data Availability

The raw data supporting the conclusions of this article will be made available by the authors, without undue reservation.
